# Ureteral Diaphragmatic Hernia Treated with Ureteral Stenting: A Case Report and Review of the Literature

**DOI:** 10.1155/2022/4866502

**Published:** 2022-02-22

**Authors:** Tateki Yoshino, Ayako Itakura, Shinnosuke Fujikawa, Tomoyuki Sugitani, Kazuo Kawakami, Emi Ishibashi, Koji Kodama, Shota Oshima

**Affiliations:** ^1^Department of Urology, Shimane Prefectural Central Hospital, Shimane, Japan; ^2^Department of Radiology, Shimane Prefectural Central Hospital, Shimane, Japan

## Abstract

Ureteral diaphragmatic hernia through diaphragmatic defects is an exceptionally rare subset of ureteral hernia with only fourteen such cases reported in English manuscripts. An 85-year-old woman was introduced to our department with right flank pain, fever elevation, and nausea. Urinalysis showed bacteriuria, and *Escherichia coli* was detected in the urine culture. Blood analysis revealed abnormal findings, including elevated WBC count (10,510/*μ*l) and C-reactive protein (0.28 mg/dl). Computed tomography (CT) of the abdomen demonstrated a defect of the right diaphragmatic crus containing a dilated right ureter with associated hydronephrosis. Retrograde pyelography showed hydronephrosis and dilated ureter loops through the defect of diaphragmatic crus, known as a “curlicue sign,” and the diagnosis was right ureteral diaphragmatic hernia. A ureteral stent was placed on her right side, and the ureter was reducted into the retroperitoneal space. After six months, the ureteral stent was removed, with no subsequent recurrence of the ureteral diaphragmatic hernia at seven months. We reviewed all cases in the literature published in English of ureteral diaphragmatic hernia. While the etiology of ureteral diaphragmatic hernia is unknown, our present case and previous reports suggest that a ureteral diaphragmatic hernia may occur due to hepatic atrophy and/or an elevated position of the right kidney.

## 1. Introduction

Ureteral hernias are a rare occurrence normally identified incidentally on imaging or during surgical hernia correction and can be a cause of ureteral obstruction [1]. These hernias can occur in several locations including the inguinal, femoral, obturator, sciatic, and thoracic regions [2]. The rarest location of a ureteral hernia is through a defect in the diaphragmatic muscle [3] with fourteen such cases reported since 1958 in the English language literature [4, 5]. Ureteral diaphragmatic hernias have been found in the the retrocrural area [2, 6] and through congenital Bochdalek foramen [3, 5, 7–14]. Clinical presentation is varied, from incidental radiographic finding to obstructive pyelonephritis and urosepsis. Management has ranged from conservative to ureteral stenting or surgical repair.

In this report, we present a case of right ureteral diaphragmatic hernia containing an incarcerated right proximal ureter with subsequent obstructive pyelonephritis. We review all cases of ureteral diaphragmatic hernia published in the literature in English and discuss optimal treatment methods and the etiology.

## 2. Case Report

An 85-year-old woman, with a medical history of hypertension, dyslipidemia, reflux esophagitis, osteoporosis, and kyphosis, was introduced to our department with right flank pain, fever elevation, and nausea. Her mental condition appeared stable. The patient's temperature was 37.7°C, pulse rate 72/min, and respiration 22/min. She was 148 cm in height, weighed 37.8 kg, with a BMI of 17.2 kg/m^2^, and suffered from kyphosis. The pharynx and bilateral tonsils were not swollen, and there was no evidence of rales of the lungs. However, right costovertebral angle tenderness was notable.

**Figure 1:**
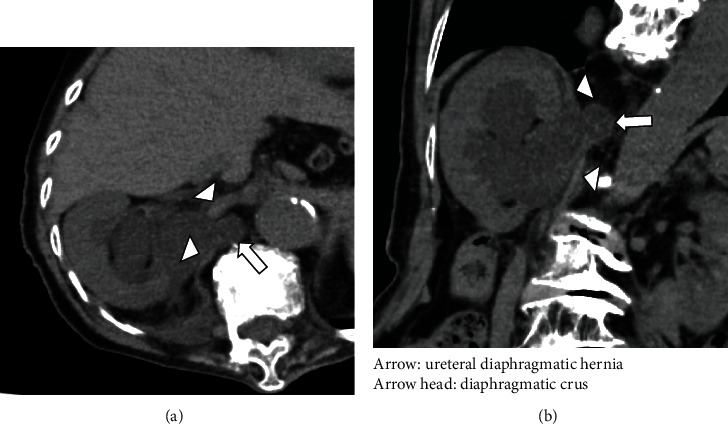
CT of the abdomen shows a diaphragmatic hernia of a part of urinary tract resulting in moderate-severe hydronephrosis ((a) axial and (b) coronal).

**Figure 2:**
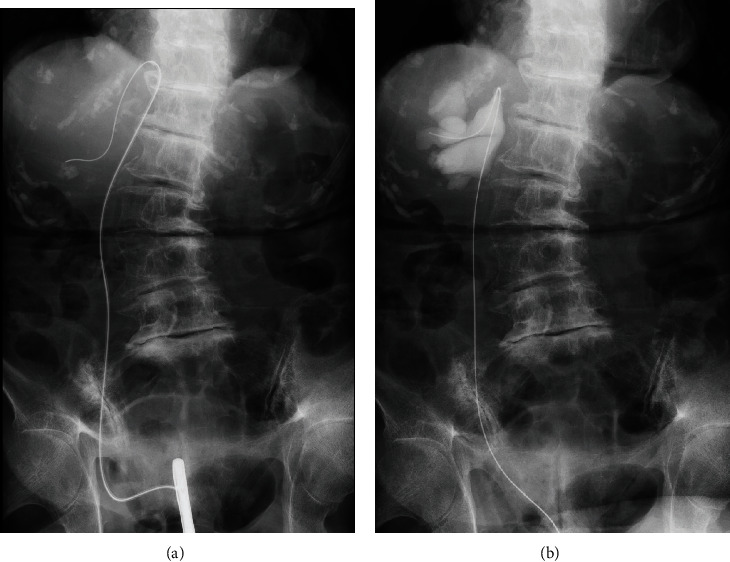
Insertion of guidewire at retrograde pyelography demonstrated ureteral loops through the diaphragm, known as a “curlicue sign” (a). At the time of ureteral manipulation by stent, the incarcerated ureter was reducted to the retroperitoneal cavity (b).

Urinalysis showed bacteriuria. *Escherichia coli* and *Streptococcus* were detected in the urine culture. Abnormal findings of a complete blood count and laboratory examination included an elevated WBC count (10,510/*μ*l) and C-reactive protein (0.28 mg/dl) and serum creatinine of 0.98 mg/dl. Ultrasound examination revealed right hydronephrosis, and subsequent CT of the abdomen revealed a right diaphragmatic hernia of a part of the urinary tract, causing moderate to severe hydronephrosis (Figures 1(a) and 1(b)); there were no signs of intraperitoneal lesions. Retrograde pyelography in combination with an inserted guidewire demonstrated ureteral loops known as a “curlicue sign” through the defect of the diaphragmatic crus, (Figure 2(a)), and our diagnosis was a right ureteral diaphragmatic hernia with associated hydronephrosis and obstructive pyelonephritis. For drainage of bacteriuria and improved renal function, a ureteral stent was placed on the patient's right side using cystoscopic guidance. At the time of ureteral manipulation by stent, the incarcerated ureter was reducted to the retroperitoneal cavity (Figure 2(b)).

**Figure 3:**
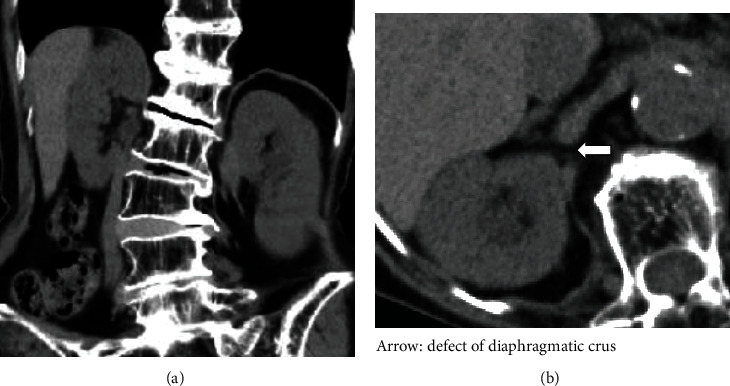
Previous CT of the abdomen revealed the right kidney in a higher position than the left one ((a) coronal) and a defect of the right diaphragmatic crus which eventually became the hernia orifice ((b) axial).

A CT done one year previously on our patient showed neither a right ureteral diaphragmatic hernia nor hydronephrosis; however, it did reveal the higher position of the right kidney due to hepatic atrophy (Figure 3(a)), and a defect of the right diaphragmatic crus, which eventually developed into the hernia orifice (Figure 3(b)**).**

Based on the diagnosis of pyelonephritis and the results of the urine culture, we administered antibiotics and placed an indwelling Foley catheter. Treatment resulted in reduced inflammation and hydronephrosis, and improvement of the ureteral diaphragmatic hernia was confirmed by CT. The stent was removed at six months after placement, and at seven months after removal, there had been no recurrence of pyelonephritis or right ureteral diaphragmatic hernia.

## 3. Discussion

It is a rarity to encounter a ureteral hernia. Pollack et al. reported that 120 reports of ureteral hernias had been confirmed at the time of their case-series publication in 1975 [15]. While the exact number of ureteral hernias is unknown, recent publications have documented less than 200 cases [10]. Common locations of ureteral hernias are the inguinal, femoral, sciatic, obturator, and thoracic regions. Ureteral inguinal hernias occur most commonly (42-64%) in these regions [15], while the rarest ureteral hernia, the ureteral thoracic hernia, occurs through a muscular diaphragmatic defect.

We conducted a retrospective review of reported cases, and published papers were retrieved from Medline, PubMed and Google Scholar. Literature review and a case report revealed 14 documented cases of ureteral diaphragmatic hernia [4, 5], making, to our knowledge, the current case the 15th such patient diagnosed and reported. Clinical data of these 15 cases were reviewed and are summarized in Table 1, and aggregate results are set out in Table 2.

A majority of the reviewed 15 cases occurred in the elderly, with the diagnosis mean age being 75.6 years; 10 (66.6%) of the 15 were female. The right side was the diseased side in all cases; some ureteral hernias presented with symptoms attributable to the ureteral hernia/urinary tract obstruction while others were detected incidentally. Symptomatic cases presented with flank pain, renal dysfunction, gross hematuria, nausea, pyelonephritis, and urosepsis. Of the 15, 8 cases (53.3%) presented with right flank pain [3, 5, 7, 8, 11, 12, 14], while 7 (46.6%) were incidental findings in a workup for azotemia [10], contralateral flank pain [9], renal [2] and ureteral stones [16], vomiting and loose stools [4], a PET-CT scan for the workup of a lung nodule [6], and one presented as septic obstructive pyelonephritis [13]. With relation to the hernia orifice, 10 cases were herniated through the right Bochdalek foramen [3, 5, 7–14] and the other 5 were located in the retrocrural region [2, 6], diaphragm [4, 16], or diaphragmatic crus.

Ten of the cases were diagnosed by CT combined with pyelography (retrograde, antegrade, and intravenous) [2, 8–14, 16], four were by CT only [3–6] and one was solely by retrograde pyelography [7]. A curlicue sign, referring to the loop or spiral configuration of the herniated ureteral segment, was identified in all cases.

Of these 15 cases, 3 (20.0%) were managed conservatively [4, 5, 10], 4 (26.6%) underwent surgical repair [2, 3, 7, 8], and 7 (46.6%) were treated by ureteral stent [3, 11–14, 16]. Two cases did not report management [6, 9]. In the last 10 years, there has been only one reported case of surgical repair [3] and the 7 cases here of ureteral stenting were also reported in that period [3, 11–14, 16]. Within the 7 ureteral stent cases, one was followed by surgical repair due to failure of ureteral reduction by stenting [3]; a second required a transient nephrostomy due to difficulties with retrograde stenting [13]; and a third case underwent intrarenal surgery with stenting [16]. Two cases required ureteral stent removal: one had no relapse of ureteral hernia [14], while the second experienced a relapse of the ureteral hernia but no urinary tract obstruction was observed [12].

In cases of obstruction and resultant pain or pyelonephritis, drainage becomes imperative. Attempt at retrograde ureteral stenting is reasonable and minimally invasive [12] and was done with notable success in the reduction of an incarcerated ureter in 6 of the 7 stented cases. As the mean age of the stented-case patients undergoing ureteral stenting was higher (84.0 years) than the overall average (75.6), minimally invasive management by ureteral stenting may be the treatment of choice by doctors caring for extremely elderly patients.

Open surgery was done in 4 of the 15 cases, two of which underwent surgical repair via the retroperitoneal approach [3, 7]. Lin et al. [3] reported that the incarcerated ureteral segment was mobilized out of the diaphragmatic defect and returned to the retroperitoneal cavity, and the diaphragmatic defect was closed using interrupted 0 silk sutures; resection and anastomosis of the redundant proximal ureteral segment and nephropexy were also done. Due to the difficulty of locating a small hernia orifice, it may be important for surgical repair to be done before an incarcerated ureter is reducted to the retroperitoneal cavity. Laparoscopic surgical repair may be more optimal than open surgery as the operative field is similar to that for retroperitoneoscopic nephrectomy.

In all 15 cases of ureteral diaphragmatic hernia, the herniation lateralized to the right-side; however, the etiology of this disorder characteristic is unknown [3].

Bochdalek hernias in adults are thought to be more prevalent on the left side than the right. It has been postulated that the liver normally obstructs any right-sided defect [17]. If there is hepatic atrophy or deformity, the liver no longer shields the diaphragm completely. Thus, the exposed diaphragm may be susceptible to right diaphragmatic herniation [17, 18]. In our present case, as hepatic atrophy was seen on CT examination, the patient likely had an exposed diaphragmatic area. In support of this, the high position of the right kidney due to hepatic atrophy and a defect of the right diaphragmatic crus which later became a hernia orifice had been identified on CT one year previously. In at least 6 (including ours) of the 15 cases (40.0%), the right kidney was in a higher position [3, 9, 10, 14, 16]. Consequently, the ureteral diaphragmatic hernia may be caused as a result of hepatic atrophy and/or high position of the right kidney.

Based on the above results, we can speculate that since the right upper urinary tract was close to the defect of the right diaphragmatic crus, increased intra-abdominal pressure may have caused the right ureter to eventually herniate through the defect.

## 4. Conclusions

Ureteral hernias are generally an exceptional occurrence, with ureteral diaphragmatic hernia being even more exceedingly rare in the literature. Imaging studies such as CT and/or pyelography are imperative for the diagnosis of ureteral diaphragmatic hernia. Management strategies depend on the clinical context. In cases of obstructive pyelonephritis or renal dysfunction, drainage is essential. A trial with retrograde ureteral stenting is reasonable and minimally invasive and has a notable success rate. While the etiology of ureteral diaphragmatic hernia remains unknown, our present case and previous reports suggest that the ureteral diaphragmatic hernia may be the result of hepatic atrophy and/or a high position of the right kidney.

## Figures and Tables

**Table 1:** Review of ureteral diaphragmatic hernia reported in the literature in English.

No.	Author	Year	Age/gender	Laterality	Presentation	Hernia orifice	Treatment
1	Swithinbank	1958	60/F	R	Right flank pain	BF	Surgical repair
2	Paterson	1989	75/M	R	Right flank pain	BF	Surgical repair
3	Chawla	1994	56/M	R	Workup for left flank pain	BF	NA
4	Catalano	1998	63/F	R	Workup for renal stones	Retrocrural	Surgical repair
5	Sukumar	2010	75/F	R	Workup for azotemia	BF	Conservative
6	Balakrishnan	2011	83/F	R	Right flank pain	BF	Ureteral stenting
7	Song	2011	75/M	R	Right flank pain	BF	Ureteral stenting
8	Osman	2012	65/M	R	Right flank pain	BF	Conservative
9	Hatzidakis	2014	86/F	R	Septic obstructive pyelonephritis	BF	PNS→ureteral stenting
10	Almeida	2015	82/F	R	Workup for lung nodule	Retrocrural	NA
11	Dru	2016	94/F	R	Right flank pain	BF	Ureteral stenting
12	Lin	2017	81/F	R	Right flank pain	BF	Ureteral stenting→surgical repair
13	Beland	2019	84/F	R	Workup for ureteral stone	DP	Ureteral stenting
14	Heidar	2019	71/M	R	Workup for vomiting	DP	Conservative
15	Current case	2021	85/F	R	Right flank pain, fever	DC	Ureteral stenting

BF: Bochdalek foramen; DP: diaphragm; DC: diaphragmatic crus; NA: not available.

**Table 2:** Aggregate results of ureteral diaphragmatic hernia including present case.

Age, years old	Median: 75.0, mean: 75.6 (range: 56-94)
Gender, case	Female: 10 (66.6%), male: 5 (33.3%)
Diseased side, case	Right: 15 (100%)
Presentation, case	Rt. flank pain: 8 (53.3%), incidental: 7 (46.6%)
Diagnosis, case	CT+pyelography (RP, IVP, AP): 10 (66.6%) CT only: 4, RP only: 1 “Curlicue sign” was identified in all cases
Hernia orifice, case	Bochdalek foramen: 10 (66.6%), others: 5 (33.3%)
Treatment, case	Conservative: 3 (20.0%) Ureteral stenting: 7 (46.6%) Surgical repair: 4 (26.6%)
